# Antioxidant Capacity and Phenolics Profile of Portuguese Traditional Cultivars of Apples and Pears and Their By-Products: On the Way to Newer Applications

**DOI:** 10.3390/foods12071537

**Published:** 2023-04-05

**Authors:** João David Teixeira, Ana Rita Soares Mateus, Claudia Sanchez, Pier Parpot, Carina Almeida, Ana Sanches Silva

**Affiliations:** 1 National Institute for Agrarian and Veterinary Research (INIAV), I.P., Rua dos Lágidos, Lugar da Madalena, 4485-655 Vila do Conde, Portugal; david.teixeira@iniav.pt (J.D.T.); anarsmateus@hotmail.com (A.R.S.M.); 2 Center for Study in Animal Science (CECA), ICETA, University of Porto, 4050-453 Porto, Portugal; 3 Faculty of Pharmacy, University of Coimbra, Polo III, Azinhaga de Stª Comba, 3000-548 Coimbra, Portugal; 4 REQUIMTE/LAVQ, R. D. Manuel II, Apartado 55142, 4501-401 Porto, Portugal; 5 National Institute for Agricultural and Veterinary Research (INIAV), I.P., 2460-059 Alcobaça, Portugal; claudia.sanchez@iniav.pt; 6 GREEN-IT—Bioresources for Sustainability R&D Unit, ITQB NOVA, 2780-157 Oeiras, Portugal; 7 Department of Chemistry, University of Minho, 4704-553 Braga, Portugal; parpot@quimica.uminho.pt; 8 Center of Biological Chemistry, University of Minho, 4704-553 Braga, Portugal; 9 LEPABE—Laboratory for Process Engineering, Environment, Biotechnology and Energy, Faculty of Engineering, University of Porto, Rua Dr. Roberto Frias, 4200-465 Porto, Portugal; 10 AliCE—Associate Laboratory in Chemical Engineering, Faculty of Engineering, University of Porto, Rua Dr. Roberto Frias, 4200-465 Porto, Portugal; 11 Associate Laboratory for Animal and Veterinary Sciences (Al4AnimalS), 1300-477 Lisbon, Portugal

**Keywords:** ultra-high performance liquid chromatography, time-of-flight mass spectrometry, phenolics, apples, pears, by-products, antioxidant capacity, fructose, principal component analysis

## Abstract

Pears (*Pyrus communis* L.) and apples (*Malus domestica* Borkh.) are two of the most popular fruits worldwide. The phenolic compounds they offer are associated with human health benefits due to their antioxidant properties. Since these fruits’ by-products are not yet fully exploited, it is important to characterize them, especially in terms of their antioxidant properties. The aim of this study was to determine the antioxidant properties of old traditional cultivars, six regional pear cultivars and five regional apple cultivars grown in the Alcobaça region (Portugal). Antioxidant capacity assays were used to evaluate the antioxidant properties. Generally, the antioxidant capacity, total phenolics content (TPC), and total flavonoids content (TFC) of fruit byproducts (both seeds and peels) were higher than the corresponding mesocarp, indicating their potential as sources of beneficial antioxidant compounds. Moreover, a UHPLC-ToF-MS method was optimized and validated in order to quantify 21 distinct phenolics in these fruit samples. The analytical method’s suitability for quantifying phenolic compounds was demonstrated by an evaluation of linearity, limit of detection, limit of quantification, precision and accuracy. This method was used to determine the phenolic composition of samples of regional (local) cultivars. The phenolics in the fruit samples with the highest concentrations were phlorizin and chlorogenic acid. Principal component analysis (PCA) was used to separate distinct fruit species while emphasizing their similarities and differences.

## 1. Introduction

It is well established that fruits are a very important part of the human diet since they provide numerous nutrients such as minerals and dietary fiber, as well as bioactive compounds [1,2]. One of the most produced fruits worldwide is the apple (*Malus domestica*), mainly in the Asian continent, with the largest orchard fields being located in China [3,4]. There are over 7000 cultivars of apples identified in the world, each with a unique set of characteristics comprising size, color, firmness, shape, texture, flavor (including sweet, sour, and bitter sensations), juiciness, aroma, and nutritional value [5]. These characteristics influence the quality of the fruit and the products they originate [6]. Pears (*Pyrus communis* L.) are also planted in abundance, and like apples, they are mainly produced in China, with over 3000 cultivars identified in this country’s repertoire [7].

Both of these fruits are known for being major sources of bioactive compounds, such as phenolic compounds [8,9]. These are considered responsible for desirable health properties such as anti-inflammatory, antibacterial and antioxidant activities [9,10], which are proven to have a significant role in the prevention of diseases such as cancer [11], diabetes [12], age-related functional decline [13], cardiovascular and neurodegenerative diseases such as Alzheimer’s [14,15,16].

Over the past few years, consumers have become more informed, and they have changed their buying and consumption habits [17]. In this line, people are developing an interest in ‘natural’ foods/products and favor these over processed products [18,19]. With the previous set of characteristics in mind, the food industry aims to develop their products, incorporating ingredients that are more close to ‘natural’ foods, rich in bioactive compounds, convenient, and largely accepted by their organoleptic properties, making fruits among the most suitable foods for this goal [20].

Since the production of fruit-based products has grown in that time period, one of the challenges that the food industry faces is the amount of waste being generated from the production of fruit-based products. This poses a major concern regarding environmental pollution [21] because large quantities of by-products are discarded without any treatment. However, these by-products (seeds and peels), are a great source of bioactive compounds (such as phenolics) [22] with interesting biological properties that can have a role on the prevention and/or treatment of emerging diseases [23].

Biodiversity is declining at an alarming rate [24,25], endangering the conservation of various species. This crisis requires careful consideration and setting of objectives in order to preserve biodiversity in the world [26,27]. One way of tackling this problem is to find uses for lesser-known species, making them targets of interest for the food industry.

Therefore, the objective of this study is to determine the antioxidant properties of five apple cultivars (*Pêro de Borbela*, *Pardo Lindo, Repinau*, *Pêro Coimbra* and *Noiva*) and six pear cultivars (*Bela-Feia*, *Torres Novas*, *Carapinheira*, *Carapinheira Roxa*, *Lambe-os-Dedos* and *Amorim*), from the Alcobaça (Portugal) region to characterize Portuguese traditional cultivars regarding these fruits’ bioactive composition, for which there is a lack of information. Moreover, the evaluation of the fructose content of both apple and pear cultivars was performed to conclude their potential to substitute sugar in food formulations. Moreover, in accordance with international guidelines, an analytical method was optimized and validated for the detection of 21 individual phenolic compounds in fruits using UHPLC-ToF-MS. PCA was used as a valid tool to distinguish between fruit species/cultivars and fruits parts. These data will allow to preserve fruits’ biodiversity and might increase the interest of both fruit growers and food industry by the exploration of some of these cultivars.

## 2. Materials and Methods

### 2.1. Cultivars under Study

Five apple and six pear cultivars from the Alcobaça region (Portugal) were collected from Vieira de Natividade Fruit Research Station (Alcobaça) of the National Institute of Agrarian and Veterinary Research (INIAV), between July and November 2021 and July and October 2022. The apple regional cultivars included were *Pardo Lindo*, *Repinau*, *Pêro de Borbela*, *Pêro Coimbra*, and *Noiva*, and the pear regional cultivars were *Carapinheira*, *Carapinheira Roxa*, *Amorim*, *Lambe-os-Dedos*, *Bela-Feia* and *Torres Novas*. Photographs of the Portuguese traditional cultivars can be found in this publication—see Supplementary Materials, Figure S1.

### 2.2. Sample Preparation

Ten to twelve fruits from each cultivar were used in this study. Each fruit was separated into three sections: peels, seeds and pulp (mesocarp). The three sections were individually homogenized with a grinder homogenizer (Ultra Turrax^®^ T25, Janke and Kunkel IKA, Germany). Two grams of the homogenized sample was weighted into a 50 mL Falcon tube and mixed with 20 mL of the selected solvent (two separate extracts were prepared—one using water as the solvent and the other using 95% ethanol) on an Ultra-TURRAX homogenizer for 3 min. The remainder of the homogenates were stored at <−20 °C.

For the UHPLC phenolic compounds analysis, a solid–liquid extraction methodology was used. For this purpose, two grams of each sample was added to 10 mL of MeOH:H_2_O:formic acid (49.95:49.95:0.10 *v*/*v*/*v*) and further sonicated at room temperature for 10 min. After this, the solution was agitated for 15 min in a horizontal shaker and centrifuged at 2250× *g* for 10 min at 20 °C. The supernatant was removed to another Falcon tube, and the extract was repeated with another 10 mL of the solvent. The second extract was merged with the first one.

### 2.3. Chemicals and Reagents

The phenolic compound standards (4-hydroxybenzoic acid, apigenin, caffeic acid, catechin, chlorogenic acid, epicatechin, eriodictyol, gallic acid, luteolin, naringenin, o-coumaric acid, *p*-coumaric acid, phloridzin, quercetin, quercetin-3-B-D-glucoside, quercitrin, rutin, sinapic acid, syringic acid, trans-ferrulic acid, and vanillic acid), (±)-6-hydroxy-2,5,7,8-tetramethylchromane-2-carboxylic acid (Trolox), 2,2-diphenyl-1-picrylhydrazyl (DPPH), Folin-Cioucalteu reagent, sodium carbonate, sodium nitrite, aluminum chloride, sodium hydroxide, β-Carotene, chloroform, Tween^®^ 40, linoleic acid, fructose, resorcinol, thiourea, and formic acid were purchased from Sigma Aldrich. Ethanol, methanol, acetic acid, and hydrochloric acid were purchased from Honeywell.

### 2.4. Evaluation of the Dry Mass Percentage

Dry mass content (%) was measured for each sample in a hot air conventional oven (Memmert, Nurenberg, Germany). Fruit samples of approximately 10 g, cut into slices, were dried at 70 ± 1 °C until they reached a constant mass, according to AOAC [28]. All determinations were made in triplicate.

### 2.5. Antioxidant Activity Assays

#### 2.5.1. DPPH Radical Scavenging Assay

The DPPH radical scavenging assay was employed as described by Martins et al. [29]. Briefly, 50 µL of sample was mixed with 2 mL of the DPPH radical solution (14.2 µg/mL) in a 15 mL Falcon tube, kept in the dark for 30 min and the absorbance was read in a spectrophotometer (UV-Vis Spectrophotometer (U-2810, Hitachi, Digilab, Sydney, NSW, Australia) at 515 nm. A calibration curve (y = 0.8457x − 3.2621, r^2^ = 0.9980) was drawn up using different concentrations of Trolox (6-hydroxy-2,5,7,8-tetramethylchroman-2-carboxylic acid), with a working range of 5–100 µg/mL. Results were expressed as µg Trolox equivalent (TE)/g of fruit. The inhibiting percentage was calculated according to the following formula:

(1) $$Inhibiting\;percentage\;\left(\%\right)=\left(\frac{Abs\;control-Abs\;sample}{Abs\;control}\right)\times 100,$$

where *Abs control* is the absorbance of the control and *Abs sample* corresponds to the absorbance of the sample. All experiments were conducted using duplicates.

#### 2.5.2. β-Carotene Bleaching Assay

This method was carried out following the procedure of Miller [30]. In this method, 1 mL of a β-carotene solution (0.2 mg/mL) in chloroform was mixed with 20 mg of linoleic acid and 200 mg of Tween^®^ 40 emulsifier. The chloroform was evaporated on a rotary evaporator at 40–45 °C. Then, 100 mL of ultrapure MilliQ^®^ water, which was vortexed for 30 min, was added, and thoroughly agitated, until an emulsion was formed. Finally, to 200 µL of each sample, 5 mL of the β-carotene emulsion was added and held for two hours at 55 °C in a water bath. Both the samples and the control absorbance were read in a spectrophotometer at 470 nm at the conclusion of this time frame. The antioxidant activity coefficient (*AAC*) was calculated resorting to the following equation:

(2) $$AAC=\left(\frac{Abs\;sample-A2\;control}{A0\;control-A2\;control}\right)\times 100,$$

where *A*0 *control* is the absorbance of the control at the initial time, *t* = 0 min, *A*2 *control* is the absorbance of the control after 120 min at 50 °C, and *Abs sample* is the absorbance of the sample after it has also been subjected to 50 °C for 120 min in a water bath. All experiments were conducted using duplicates.

### 2.6. Total Phenolics Content (TPC) Assay

In order to estimate the total phenolics content of the fruit extracts, the method by Erkan et al. [31] was applied. This method consists of mixing 1 mL of sample with 7.5 mL of Folin–Cioucalteu reagent (10% *v*/*v*) and letting it react for 5 min, before adding 7.5 mL of Na₂CO₃ (60 mg/mL) and reading the absorbance, after 120 min at 725 nm. A calibration curve (y = 0.0065x − 0.0057, r^2^ = 0.9997) was drawn up using different concentrations of gallic acid with a working range of 5–150 µg/mL. Results were expressed as µg Gallic acid equivalents (GAE)/g of fruit. All experiments were conducted using duplicates.

### 2.7. Total Flavonoids Content (TFC) Assay

The total flavonoid content method was performed according to Barbosa et al. [32]. In this assay, 1 mL of sample was mixed with 4 mL of ultrapure water and 0.3 mL of sodium nitrite (50 mg/mL). After 5 min, 0.6 mL of aluminum chloride (100 mg/mL) was added to the mixture and after another 6 min, 2 mL of sodium hydroxide (40 mg/m) and 2.1 mL of ultrapure water were added. The absorbance of samples was then read in a spectrophotometer at 510 nm. A calibration curve (y = 0.0017x + 0.0165, r^2^ = 0.9986) was drawn up using different concentrations of Epicatechin, with a working range of 5–150 µg/mL. Results were expressed as µg Epicatechin equivalents (EE)/g of fruit. All experiments were conducted using duplicates.

### 2.8. Fructose Content Assay

The total amount of fructose present in the samples was assessed using the method described by Ashwell [33]. For the performance of this method, resorcinol reagent was prepared by mixing 1 g of resorcinol with 250 mg of thiourea in 100 mL of glacial acetic acid. Then, 2 mL of sample was mixed with 1 mL of the resorcinol reagent, and 7 mL of a diluted hydrochloric acid solution was added to the mixture. The sample mixtures were kept in a water bath at 80 °C for 10 min. At the end of this period, all samples were cooled under tap water for 5 min and read in a spectrophotometer at 520 nm. A calibration curve (y = 0.0016x + 0.0524, r^2^ = 0.9995) was drawn up using different concentrations of fructose, with a working range of 5–500 µg/mL. Results were expressed as mg fructose/g of fruit. All experiments were conducted using duplicates.

### 2.9. UHPLC-ToF-MS Conditions

The detection and quantification of the phenolic compounds were performed using a Nexera X2 Shimadzu UHPLC coupled with a 5600+ ToF-MS detector (SCIEX, Foster City, CA, USA) equipped with a Turbo Ion Spray electrospray ionization source working in positive mode (ESI+). Regarding the analytical column, a Acquity UPLC BEH C18 (2.1 mm × 100 mm, 1.7 μm) was used. The column temperature was kept at 20 °C, and the autosampler was maintained at 20 °C. The chromatographic separation took place in gradient mode using an aqueous solution of 0.1% formic acid (eluent A) and methanol with 0.1% formic acid (eluent B) as the mobile phase. The injection volume for both standards and samples was 20 µL. The selected gradient program was the following: 0–0.5 min kept at 90% [A]; 0.5–8 min from 90% to 20% [A] and kept until the end of the run at 20% [A], completing a total run time of 8.1 min. Using the Analyst^®^ TF 1.7 software (SCIEX, Foster City, CA, USA) and the following parameters for mass spectrometry, the acquisition was carried out in full scan from 100 to 750 Da: ion source voltage of 5500 V; source temperature of 575 °C; curtain gas (CUR) of 30 psi; Gas 1 and Gas 2 of 55 psi; and declustering potential (DP) of 100 V. To provide accurate mass resolution, the ToF-MS detector was calibrated every 7 injections in the method’s mass range.

PeakView™ 2.2 and MultiQuant™ 3.0 software (SCIEX, Foster City, CA, USA) were used for phenolic compound identification and data processing. PeakView™ 2.2 software automatically presents the isotope match. Two parameters and their accompanying equations (Equations (3) and (4)) were employed for phenolic compound identification: (1) maximum retention time deviation (∆RRT) of 0.1 min (Equation (3)); and (2) exact mass deviation (m) with a tolerance of 5 ppm (Equation (4)).

(3) $$\mathrm{RRT}=\left(\frac{\mathrm{RRT}\;\mathrm{spiked}\;\mathrm{samples}\;-\;\mathrm{RRT}\;\mathrm{standard}}{\mathrm{RRT}\;\mathrm{standard}}\right)\times 100$$

(4) $$\Delta m\;\left(\mathrm{ppm}\right)=\left(\frac{\mathrm{Exact}\;\mathrm{mass}\;-\;\mathrm{Detected}\;\mathrm{mass}}{\mathrm{Exact}\;\mathrm{mass}}\right)\times 10^{6}$$

where RRT is the relative retention time and ∆m is the exact mass deviation.

### 2.10. Validation of the UHPLC-ToF-MS Method

The method was validated by the determination of the working range, linearity, limit of detection (LOD), limit of quantification (LOQ), precision and accuracy (through recovery assays). LOD and LOQ were determined as the concentration that originates a signal-to-noise ratio (S/N) ≥3 and ≥10, respectively. According to Directive 96/23/EC, the trueness of measurements can be assessed through the recovery of additions of known amounts of the analytes to a blank matrix, when certified reference materials are not available.

### 2.11. Principal Component Analysis (PCA)

Principal component analysis, classification and regression by machine learning were carried out using Python (Python 3).

### 2.12. Statistical Analysis

To evaluate the effect of the harvest year, cultivar, and part of the fruit on the antioxidant properties and phenolic profile of the samples a two-way analysis of variance (ANOVA) was performed. Significance was defined at least at *p* < 0.05. All data analyses were carried out using Microsoft Excel 365 equipped with Analysis ToolPak.

## 3. Results and Discussion

### 3.1. Dry Mass Percentage Assay

Measurement of the dry mass content led to the information presented on Table 1. The observed percentages are similar to the ones reported by Instituto Nacional de Saúde Dr Ricardo Jorge (INSA), 17.1% for apple [34] and 14.9% for the mean of five cultivars of pear [35]. The old traditional Portuguese cultivars of apples showed dry mass contents between 18.3 and 22.8% while the pear cultivars exhibited values held between 16.7 and 20.7%.

### 3.2. Antioxidant Capacity Assays

Four tests were conducted to determine the antioxidant properties of the fruit cultivars. The DPPH radical scavenging assay and the β-carotene bleaching assay were used to measure the antioxidant capacity. The other two tests evaluated the content of compounds that contribute to the antioxidant properties. These tests consisted of the total phenolic content (TPC) assay and the total flavonoid content (TFC) assay. The tests were repeated for two harvests of each cultivar, 2021 and 2022, thus analyzing if the time frame can be considered a factor that originates differences in the amount of phenolic compounds present in the fruit. Other possible factors are the edaphoclimatic conditions, the cultivar itself, or the pesticide/fertilizer appliance.

Table 2 and Table 3 show the inhibiting percentage (IP) and AAC obtained by all the cultivars of apples and pears, respectively.

The *Noiva* cultivar presented the highest IP, in the peel portion (53.4 ± 1.00 and 51.5 ± 1.96% in 2021 and 2022, respectively), followed by the *Pêro de Borbela* (27.8 ± 0.61 and 39.7 ± 0.87% in 2021 and 2022, respectively). However, the seed portion of the 2022 harvest of the *Pêro de Borbela* cultivar presented a higher IP (14.6 ± 1.09%) than the ones presented by both harvests of the *Noiva* cultivar (13.3 ± 0.18 and 11.5 ± 0.65%). One common point among all the apple cultivars is that the by-products present a higher IP than the pulp, in which the maximum registered IP among edible portions was 6.15 ± 0.44% for the *Noiva* cultivar. According to the DPPH radical scavenging inhibition assay, the *Noiva* and *Pêro de Borbela* cultivars stand out among all the apple cultivars that were examined in terms of their antioxidant capacity, mostly in the by-products. These findings are comparable to those published by Moni Bottu et al. [10] who found that using a similar solvent (MeOH:H_2_O 80:20) and reporting the results in fresh weight (FW), the tested apple cultivars, *Bittersweet* and *Jonagold*, obtained an IP of 14.4 ± 1.8% and 13.4 ± 3.1%, respectively. These results, however, represent the whole fruit instead of a portion of it. The same problem was registered in the work of Mignard et al. [5], where among all the 155 analyzed cultivars, the minimum and maximum relative antioxidant content identified were 1.7 and 44.6 mg Trolox/100 g FW. In the present work, if we convert our results into mg Trolox/100 g of FW, we obtain values ranging from 3.34 to 64.70, meaning that the studied cultivars possess similar antioxidant properties to those studied by the authors.

The *Noiva* cultivar excelled all other cultivars regarding the AAC value registered, in both by-products (283 ± 3.62 and 270 ± 8.83 in the peel and 196 ± 7.24 and 122 ± 6.62 in the seed) but not in the pulp. The second highest AAC was registered in the peel of the *Pêro de Borbela* cultivar (245 ± 4.41). All the AAC values for pulp were very similar to each other, with the results being held between 51.5 ± 4.41 (*Repinau*) and 87.4 ± 6.64 (*Pêro de Borbela*). These findings are in agreement with the results reported by Sara et al. [36], and in some cases, such as in the *Noiva* by-products, our results showed higher antioxidant capacity than the cultivars presented by other authors.

Regarding the pear cultivars, the *Amorim* and *Bela-Feia*, as well as the *Carapinheira* (of which we were only able to obtain data from the 2021 harvest) cultivars can be highlighted regarding the IP they present in the seed portion, 15.4 ± 1.31 and 13.7 ± 1.89%, 13.2 ± 0.31 and 15.8 ± 0.89%, and 14.8 ± 0.00%, respectively. In contrast to the apple cultivars, pear seeds present, in some cases, significantly higher IP than the peel portion of the same fruit. The *Carapinheira* cultivar is the greatest example of this, showing an IP of 21.4 ± 0.15%, but the *Amorim* 2022 and *Torres Novas* 2021 harvests present the same profile regarding this parameter. The IP on the pulps of pear cultivars is also interesting to analyze because according to the DPPH radical assay, the mesocarp of all the samples under study has a similar level of antioxidant capacity, nevertheless with significant differences. The *Amorim* pear cultivar outperforms all other cultivars, registering an IP of 8.57 ± 0.46 and 8.19 ± 0.89%, over 200 percent higher than all the other cultivars. The *Torres Novas* cultivar has not shown any IP across the two harvests. Kolniak-Ostek et al. [9] studied the antioxidant activities of different portions of the *Radona* pear cultivar, reporting DPPH radical scavenging assay values of 1210.1 ± 17.8, 1632.7 ± 18.5, and 426.0 ± 5.9 µmol TE/100 g for the peels, seeds and pulp, respectively. These can be considered very similar to the results we obtained—the maximum value in the same unit of measurement was 1438.7 ± 25.5 µmol TE/100 g DW (*Amorim* Peel) In another study, Guan et al. [37], measured the antioxidant activity of 22 Asian pear cultivars, including *Niitaka* and *Xuehua*, and found IP’s between 7.08% and 31.5%.

In general, the by-products present significantly higher AAC values than the pulp. Once again, the *Amorim* pear cultivar outperformed all the other cultivars, registering an AAC of 216 ± 7.24 and 228 ± 2.21 in the peel portion. However, the *Lambe-os-Dedos* cultivar shows a value of 60.2 ± 7.24 in the pulp, representing a higher AAC than the seed portion (39.7 ± 10.9). No pulp was able to reach AAC values close to those presented by the peel portion. This is in line with the research of Oaldje-Pavlovic et al. [38], where the authors indicate that pear peel extracts have more potent antioxidant activity than pear pulp extracts.

Koleva et al. [39] reported that the β-carotene bleaching assay is restricted to less polar compounds, whereas the DPPH radical scavenging assay is unaffected by sample polarity. This could be an indicator of why we achieved better results regarding the measured antioxidant activity with the DPPH scavenging assay than with the β-carotene bleaching assay.

Table 4 and Table 5 present the TPC (in µg of Gallic Acid Equivalents (GAE) per gram FW) and TFC (in µg of Epicatechin Equivalents (EE) per gram of FW) found for the different cultivars of apples and pears, respectively.

The TPC and TFC assays allow to conclude there is a separation between the content in phenolics and flavonoids in the three portions of the apple fruits. In all the cultivars, the peels presented a higher level of both classes of compounds, followed by the seeds and finally the pulp. Among all the by-products, the peel of the *Noiva* cultivar revealed the highest content of phenolics (1964 ± 0.00 µg GAE/g), followed by the *Pêro de Borbela* peels (1651 ± 43.7 µg GAE/g) and *Pêro Coimbra* peels (1207 ± 27.3 µg GAE/g). The other two cultivars presented similar TPC, although their content is significatively different. The seeds portion of the regional cultivars presents the same profile as the peel portions; however, the TPC values are much closer to each other across the cultivars. In this portion of the fruit, the *Pêro Coimbra* cultivar registered the highest TPC (875 ± 8.74 µg GAE/g). Across the two years, the TPC and TFC values of all the portions are significantly different, meaning that the edaphoclimatic conditions may have an important role in the amount of phenolic compounds that are produced by each cultivar. In 2012, Ceymann et al. [40] measured the polyphenol profile of 104 European apple cultivars, reporting TPC values ranging from 520 to 3790 µg GAE/g FW, meaning that the regional cultivars we assessed in this study have similar phenolic content to those analyzed by the authors.

Regarding the flavonoid content, the *Noiva* cultivar has the highest TFC in the peels (1390 ± 24.8 µg EE/g), although the *Pêro de Borbela* cultivar has higher amount in the seeds (497 ± 4.14 µg EE/g). Statistical analysis indicated there are significant differences in the TFC in the pulp portion. Gulsunoglu et al., while studying the effect of fermentation with a specific fungus on the enhancement of the phenolic compound concentration in apples [41], reported a control TFC value of <1500 µg CE/g DW (catechin equivalents per gram of dry weight), and Mignard et al. [5] reported values ranging from 7 to 1421 µg CE/g FW when assessing the antioxidant traits of 155 apple cultivars. These authors, however, used catechin as the standard for the TFC assay, thus providing the results in catechin equivalents rather than epicatechin equivalents, making the comparison with our work more challenging.

Concerning pear cultivars, the *Carapinheira* cultivar constitutes an exception to the portions´ profile displayed throughout the samples. The TPC and TFC values are significantly greater in the seeds’ portion (1164 ± 39.3 µg GAE/g and 448 ± 8.28 µg EE/g, respectively) than in the peels´ portion (1102 ± 35.0 µg GAE/g and 371 ± 24.8 µg EE/g). Among all the studied samples, this is the only cultivar in which this is registered. However, *Carapinheira* is not the pear cultivar that presents the highest TPC in the peels (the *Amorim* cultivar has a TPC of 1409 ± 40.4 µg GAE/g). A TPC value of 701 ± 7.94 µg GAE/g for the whole fruit was published by Sinha et al. [42] when studying the effects of the preservation of antioxidant properties in pears under cold storage, although in whole fruit. Furthermore, Zhou et al. [43] reported a TPC of 207 ± 13.7 µg GAE/g in the pulp of *Packham* pears. This represents a value held between the TPC values interval that the pulp of the assessed cultivars achieved in this study (170 ± 30.6–376 ± 4.37 µg GAE/g). 

Regarding the TFC, the *Torres Novas*, *Carapinheira Roxa* and *Lambe-os-Dedos*, did not register any content of flavonoids, in both harvests, in the pulps. Nevertheless, the other three cultivars presented TFC values ranging from 5.7 ± 12.4 to 61.3 ± 0.00 µg EE/g. Guan et al. [37] reported the total flavonoid content in the pulp of 22 Asian cultivars of pear, with contents held between 23.10 and 104.28 µg quercetin equivalents/g. In the by-products, the TFC was significantly higher. The peels of the *Amorim* cultivar presented the highest TFC, at 547 ± 33.1 µg EE/g. However, Wu et al. [44] reported TFC´s from 3124.6 to 6216.6 µg rutin equivalents/g of fruit. Because the total flavonoid content is expressed in different units in different scientific studies, it is very difficult to compare the results. The presentation of phenolic compounds in the scientific literature would benefit greatly from standardization or, at the very least, more harmonization [45].

### 3.3. Fructose Content Assay

Table 6 displays the results for the total fructose content of tested apple and pear samples.

The analysis of the fructose content shows that both the pulp and the by-products possess a very similar level of this sugar. However, the pulps of the *Pardo Lindo*, *Pêro de Borbela* and *Repinau* present the three highest fructose contents among all the portions and cultivars (110 ± 2.85 and 107 ± 5.05, 107 ± 4.17 and 102 ± 2.55, 101 ± 4.61 and 97.7 ± 3.95 mg/g, respectively). It also allows us to say that apple cultivars have a higher content than pear cultivars in all the portions, reporting values held between 71.3 ± 5.71 and 110 ± 2.85 mg/g, except for the *Amorim* pear seed (87.6 ± 2.41 and 88.4 ± 3.51 mg/g FW). The lowest levels of fructose in the pear cultivars, were registered by the pulp of the *Torres Novas* cultivar (39.2 ± 4.61 and 44.3 ± 3.07 mg/g), followed by the peels of the *Carapinheira* and *Bela-Feia* cultivars (46.8 ± 3.95, 47.7 ± 2.20 and 48.3 ± 3.95 mg/g, respectively).

### 3.4. Validation of the Analytical Method

Linearity was assessed using solvent calibration curves in different ranges for various phenolic compounds. Calibration curves with at least five calibration points were plotted for the studied phenolic compounds. The determination coefficients (r^2^) were always higher than 0.9905 (except for 4-hydroxibenzoic acid, gallic acid, vanillic acid), highlighting that the selected ranges are suitable to the quantification of the selected 21 phenolic compounds. Table 7 presents the results of linear range, LOQ, LOD, retention time, and recovery (at two spiking levels) for the analyzed phenolic compounds. Recovery between 80 and 120% is considered acceptable, however, if the recovery percentage is over 70%, results can be considered satisfactory. In this perspective, the recovery of the different phenolics (70.2–97.3%) is acceptable, and the extraction process is suitable for the extraction of most of the phenolic compounds from apple and pear fruit matrices.

Table 8 displays the results of repeatability and inter-day precision for the various phenolic components in a blank apple sample spiked at two different concentration levels. The method’s repeatability was assessed using the relative standard deviation (RSD_r_) for all phenolics, using the same sample and operator in a short period of time. The relative standard deviation (RSD_R_) was used to assess the method’s intra-day precision over three separate analysis days, two different concentration levels, and multiple operators.

The values obtained for repeatability are acceptable, ranging from 0.74 to 22.5%, referring to Apigenin and Naringenin, respectively. The reported values for the inter-day precision can also be considered acceptable even though the highest RSD_R_ was 23.8% (caffeic acid).

### 3.5. Quantification of Individual Phenolic Compounds

A total of 18 different phenolic compounds were identified in apple samples, but only 16 were quantified. In the pear samples, 19 phenolics were detected, of which 18 were quantified. Table 9 and Table 10 summarize the findings from these samples. As may be seen, chlorogenic acid is the most abundant phenolic in both fruits, followed by phlorizin and epicatechin. In the pear cultivars, caffeic acid is very predominant in the samples, while not present at all in the apple cultivars. Syringic acid was another phenolic compound strictly detected in the pear samples. From the 21 analyzed phenolics, apigenin and luteolin were not quantified in any of the fruit cultivars. Apigenin was detected in apples, but at levels lower than LOQ (0.0025 µg/mL). As far as the authors are aware, this is the first study to report the presence of Apigenin and Luteolin in apples and pears, although at trace levels, and quantify 4-Hydroxybenzoic acid, Eriodyctiol and Naringenin in both fruits.

Regarding the apple portions, by-products present much higher phenolic concentration when compared to the edible part of the same cultivar. These results are in agreement with the findings of Savikin et al. [46] in which the authors highlight that higher contents were found in the peel of four apple cultivars. There is no direct relation between the part of the apple fruit and the content of phenolic compounds, among by-products, since in two of the cultivars the total content was higher on seeds and in the other three cultivars, on peels. The highest phenolic content was found in the seeds of the *Pêro de Borbela* cultivar, accumulating a total of 782.6 µg/g FW, most of which were represented by phlorizin (318.7 µg/g FW), followed by chlorogenic acid (293.5 µg/g FW) and epicatechin (59.31 µg/g FW). Phlorizin has been reported to have a variety of bioactivities. In fact, phlorizin is currently extracted from some apple pomace to be used preventing obesity, and suppressing bacterial development [47,48]. These phlorizin levels are higher than those published by Karaman et al. [49] and Mihailovic et al. [50], at −23 to 159 µg/g FW and 207 µg/g FW, respectively. Chlorogenic acid levels were also higher than others previously reported in the literature. Bílková et al. [51] studied the benefits of ultra-low oxygen conditions in long-term storage and reported a chlorogenic acid content of 1.4 to 99.6 µg/g FW. The authors reported that the analysis was made just after harvesting of the fruits, which makes the results very comparable to ours. In our case, we divided the fruits in three portions, while Bílková et al. used the whole fruit. Raudone et al. [52] also revealed the phenolic content of six apple cultivars, where the chlorogenic acid levels ranged between 250 and 2000 µg/g of dry fruit. If we take into consideration the dry mass percentage of our cultivars (Table 1), we can conclude that the studied cultivars contained between 153.2 and 675.3 µg/g of dry fruit of chlorogenic acid, representing similar levels, although lower than those mentioned by the previous authors.

Regarding pear cultivars, the distribution of the phenolics among the fruit parts was very well outlined, the peels were the part where the major concentration of phenolics was found, followed by the seeds and finally the pulp. Cui et al. [53] reported the same profile for 14 Asian pear cultivars when analyzing one of the major phenolics in pears, arbutin, and Brahem et al. [54] revealed higher content of phenolics in the peel of 19 pear cultivars, in comparison to the pulp, supporting our finding.

As mentioned before, caffeic and syringic acid were quantified in the pear cultivars, and not on the apple cultivars. Caffeic acid was determined to be one of the most present phenolics in the pear samples, especially in the peels portion, where the concentration of this phenolic compound was up to 27.09 µg/g FW. This represents much higher levels of caffeic acid than the ones published by Colaric et al. [55] in *Williams* pear whole fruit (0.86 µg/g FW). The major phenolic compound in pear fruits, is however, chlorogenic acid, ranging from 21.4 to 340 µg/g FW, across the different portions. Salta et al. [56] studied the phenolic composition of five pear cultivars, including *Rocha* pear. The authors presented chlorogenic acid levels of 43–79 µg/g FW for the other four cultivars and 624 µg/g FW for the *Rocha* pear (whole fruit), meaning that our samples were very close to most of the studied cultivars.

### 3.6. Results of the Principal Component Analysis

**Figure 1:**
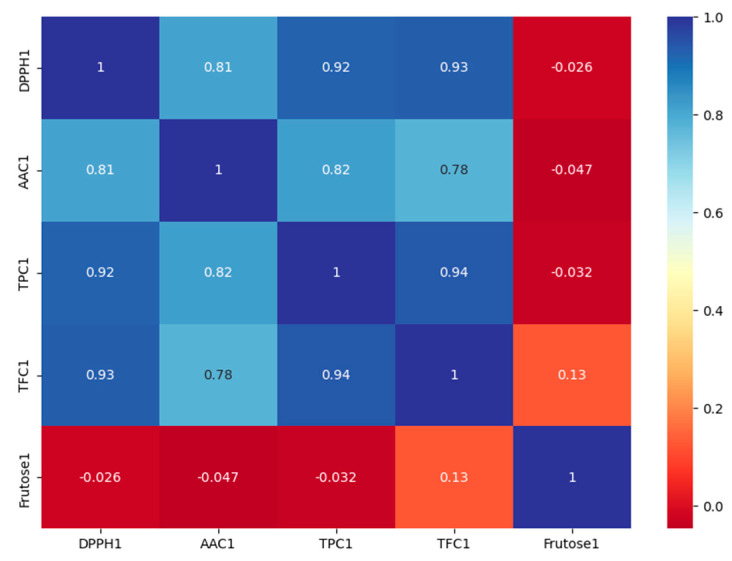
Correlation plot among the studied parameters.

The principal component analysis (PCA) is a mathematical process that employs an orthogonal transformation to turn a set of potentially correlated observations into a set of values of linearly uncorrelated variables known as principal components. The major goal is to reduce the number of dimensions while maintaining the contribution of all starting variables in order to recognize visual patterns. The correlation plot (Figure 1) shows that the fructose content is independent from all other parameters and that the TPC, TFC and DPPH radical inhibition are strongly correlated.

**Figure 2:**
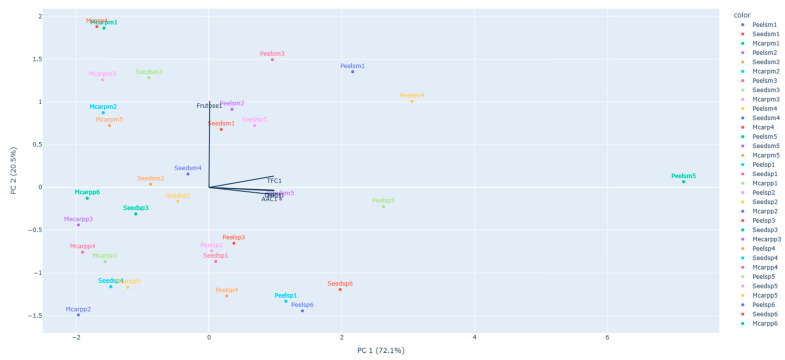
Biplot PC2 vs. PC1 for different apple and peer varieties and for the different parts of the fruits. (Peelsm1: Peel of *Pêro Coimbra* Apple, Seedsm1: Seeds of *Pêro Coimbra* Apple, Mcarpm1: Pulp of *Pêro Coimbra* Apple, Peelsm2: Peel of *Pardo Lindo* Apple, Seedsm2: Seeds of *Pardo Lindo* Apple, Mcarpm2: Pulp of *Pardo Lindo* Apple, Peelsm3: Peel of *Repinau* Apple, Seedsm3: Seeds of *Repinau* Apple, Mcarpm3: Pulp of *Repinau* Apple, Peelsm4: Peel of *Pêro de Borbela* Apple, Seedsm4: Seeds of *Pêro de Borbela* Apple, Mcarpm4: Pulp of *Pêro de Borbela* Apple, Peelsm5: Peel of *Noiva* Apple, Seedsm5: Seeds of *Noiva* Apple, Mcarpm5: Pulp of *Noiva* Apple, Peelsp1: Peel of *Bela-Feia* Pear, Seedsp1: Seeds of *Bela-Feia* Pear, Mcarpp1: Pulp of *Bela-Feia* Pear, Peelsp2: Peel of *Torres Novas* Pear, Seedsp2: Seeds of *Torres Novas* Pear, Mcarpp2: Pulp of *Torres Novas* Pear, Peelsp3: Peel of *Carapinheira Roxa* Pear, Seedsp3: Seeds of *Carapinheira Roxa* Pear, Mcarpp3: Pulp of *Carapinheira Roxa* Pear, Peelsp4: Peel of *Lambe-os-Dedos* Pear, Seedsp4: Seeds of *Lambe-os-Dedos* Pear, Mcarpp4: Pulp of *Lambe-os-Dedos* Pear, Peelsp5: Peel of *Amorim* Pear, Seedsp5: Seeds of *Amorim* Pear, Mcarpp5: Pulp of *Amorim* Pear, Peelsp6: Peel of *Carapinheira* Pear, Seedsp6: Seeds of *Carapinheira* Pear, Mcarpp6: Pulp of *Carapinheira* Pear).

PCA uses eigenvectors to transform the original variables into new ones called principal components, which are determined by assigning a coefficient to each original variable proportional to their contribution to this transformation (rotation) in order to maximize the variances of the first few components. The biplot graph (Figure 2), which projects the samples (scores) and the variables (loadings) onto a two- or three-dimensional graph, demonstrates first how the samples are related to one another and second how each variable affects each sample.

These two PCs explain 95% of the observed variation in the original data. It can be noticed that the peel of the *Noiva* variety has a very distinct set of characteristics compared to the other portions. We can also see that the fructose content is the main differentiator between apple and pear portions. TFC, TPC and DPPH radical inhibition are the main factors that contribute to the separation of the pulp and by-products in the biplot chart. This graph confirms also the correlation between these last variables.

#### 3.6.1. Principal Component Analysis

**Figure 3:**
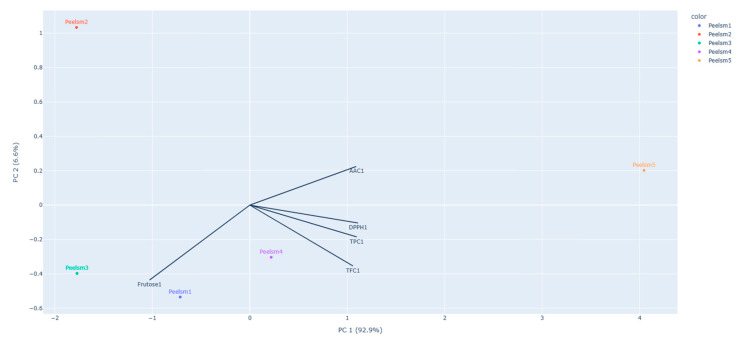
Biplot PC2 vs. PC1 for the peel moiety of the different apple varieties. (Peelsm1: Peel of *Pêro Coimbra* Apple, Peelsm2: Peel of *Pardo Lindo* Apple, Peelsm3: Peel of *Repinau* Apple, Peelsm4: Peel of *Pêro de Borbela* Apple, Peelsm5: Peel of *Noiva* Apple).

PCA performed for the individual apple peels, which explains 99% of the variation of the original data (Figure 3), shows that the *Pêro Coimbra*, *Repinau* and *Pêro de Borbela* peels are the most similar among the group. *Noiva* and *Pardo Lindo* peels are detached from the others, meaning that they possess the most singular profile. The fructose content affects strongly the peels of Pêro Coimbra and Repinau apple varieties, while TFC has higher effect on Peel of Pêro de Borbela specie. The graph shows also that the fructose content and the parameter AAC are inversely proportional.

#### 3.6.2. Classification with Machine Learning Algorithms

**Figure 4:**
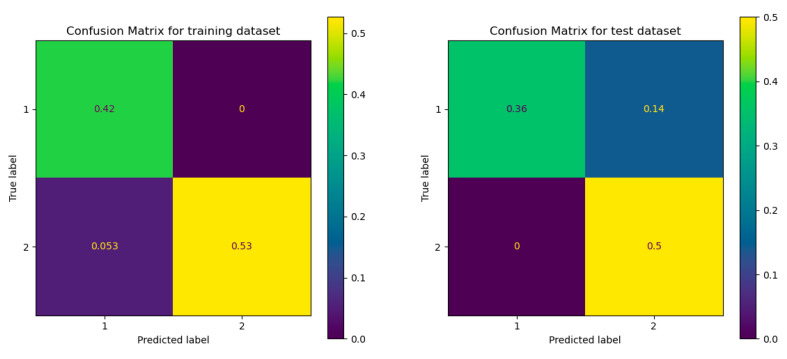
Confusion matrix of the training group (at the (**left**)) and the test group (at the (**right**)).

The availability of multivariate data matrices makes it feasible to classify individuals and rapidly extract the most crucial information from data using statistical and mathematical methods. In order to place new, unknown samples in one of the recognized classes based on their measurement pattern, supervised pattern recognition techniques use information about the samples’ membership in a given group (class or category). In this case, there were two types of samples: apples and pears. Supervised pattern recognition requires a training set of samples that fall into recognized categories in order to create a model for the classification of the data. The decision tree (DT) classifier was used, and the data were divided into a training (60%) and a test group (40). The scores for the training and the test groups were, respectively, 0.9474 and 0.8571, indicating that the algorithm provides a satisfactory classification. The confusion matrix which shows how often a sample that belongs to a given class was classified as belonging to the other, were given in Figure 4. for the training and the test group. The results show that only a few percentages of the samples are incorrectly classified.

**Figure 5:**
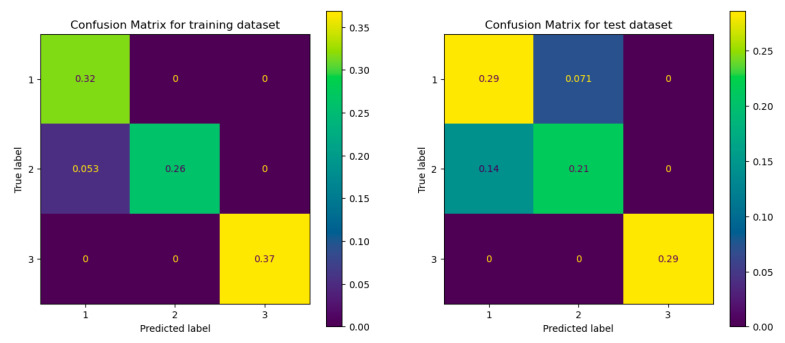
Confusion matrix of the training and test groups, regarding the samples’ membership to one of three portions.

The same principle was applied to the samples’ membership to one of three portions (peels, seeds and pulp). The scores corresponding to the training and the test groups were, respectively, 0.9474 and 0.7857. Although the results are less satisfactory than those found for the previous case, the score corresponding to the test group indicating a relatively good model fit remains acceptable. The confusion matrix given in Figure 5 shows that the highest number of false positives corresponds to the second class.

**Figure 6:**
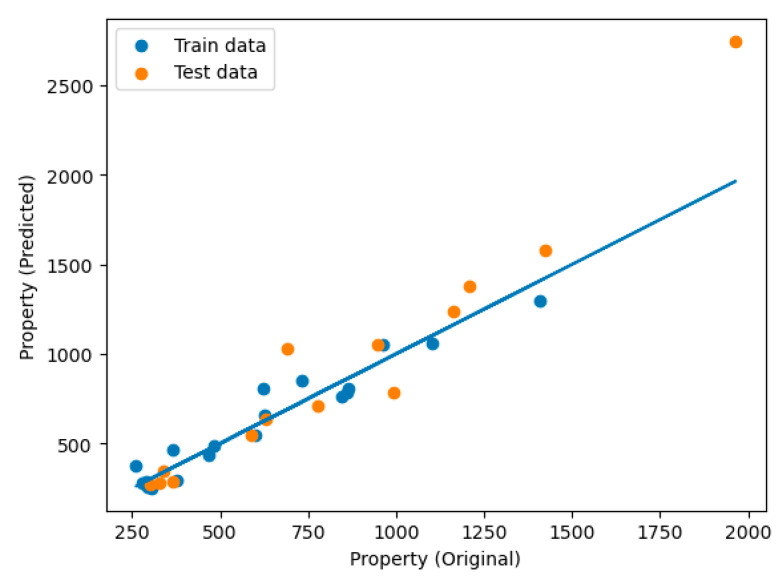
Representation of the results of the regression model created to predict the TPC in different fruit parts.

Another use for machine learning could be the prediction of how one of the variables is going to behave when the rest of them were known. In this study, the TPC of different parts of distinct cultivars of apples and pears was assessed using Linear Regression regressor. The results (Figure 6) indicate that the regression model was able to predict the total phenolics content of the specified sample with a coefficient of determination of 0.93 for the training group and 0.73 for the test group. 

The regression can be also applied to predict TFC. The scores for training and test groups in this case are, respectively, 0.8754 and 0.8491.

## 4. Conclusions

Fruit consumption is essential for a well-balanced diet, healthy habits, and illness prevention. In fact, in order to achieve these nutritional and health benefits, The World Health Organization (WHO) suggests eating at least 400 g daily, more or less five pieces [57]. Such information even originated popular sayings as: “One apple a day, keeps the doctor away”. Yet, since only around 70% of the fruit is edible, there are a lot of by-products produced from these fruits. Reusing fruit by-products is both necessary and advantageous because they contain a significant amount of phenolic compounds. Thus, this generates increasing interest from the food industry because it turns the otherwise considered waste, into material with the potential to be used with other applications such as functional foods and/or active packaging, towards a circular economy. Another important aspect to consider from this study is the need to guarantee that biodiversity continues to exist. If we can prove that regional, i.e., less well-known and less-produced cultivars of fruits, have attributes that will increase their production at a large scale, we can predict that these fruits will generate a large amounts of waste (by-products).

This study successfully established an analytical method for the determination of phenolic compounds in fruit pulps and by-products using ultra-high performance liquid chromatography coupled with time-of-flight mass spectrometry. The method was applied to thirty-three samples of regional fruits. The limits of quantification obtained were all lower than 10 µg/g in the case of phenolic acids and 2.5 µg/g in the case of other phenolics. Recovery tests showed values between 70 and 96% for the lower fortification level (0.1 mg/100 g) and 75 and 97% for the higher fortification level (1.0 mg/100 g).

The antioxidant capacity of the regional cultivars of apples and pears was evaluated and compared. In general, apple cultivars present a higher content of total phenolics and total flavonoids, as well as a higher antioxidant capacity.

The multivariate analysis was used in order to classify different apple and pear varieties and separate them into several groups. Another goal was to study the effect of different parameters on the definition of chemical properties of the samples. PCA was performed separately for the whole fruit samples and for the apples alone with total cumulative variances of, respectively, 93 and 99% corresponding to the first two PCs. These results demonstrate that multivariate analysis, with a particular emphasis on PCA, can be a useful technique for separating various fruit species while highlighting their similarities and differences. Supervised machine learning algorithms were also used for classify and predict the value of a given parameters with satisfactory outcome. In this context, the regression model was able to predict the total phenolics content of the specified sample with a coefficient of determination of 0.73 for the test group.

Although a very descriptive analysis of the antioxidant properties and total phenolics, flavonoids and fructose content were performed, in the future, it would be interesting to evaluate also possible contaminants of these samples such as mycotoxins and pesticides residues, to ensure food safety when reusing the fruits by-products.

## Figures and Tables

**Table 1:** Dry mass percentage of apple and pear cultivars. Results are expressed in means ± standard deviation.

Fruit	Cultivar	Dry Mass Percentage
Apple	*Pêro Coimbra*	18.8 ± 0.4
	*Repinau*	20.8 ± 0.4
	*Pardo Lindo*	22.8 ± 0.5
	*Pêro de Borbela*	18.6 ± 0.1
	*Noiva*	18.3 ± 0.7
Pear	*Bela-Feia*	20.2 ± 1.4
	*Torres Novas*	17.3 ± 1.0
	*Carapinheira Roxa*	20.5 ± 0.3
	*Lambe-os-Dedos*	16.7 ± 1.0
	*Amorim*	20.7 ± 1.5
	*Carapinheira*	19.7 ± 0.5

**Table 2:** Antioxidant capacity of different portions of apple cultivars using two methods: DPPH radical scavenging assay and the β-carotene bleaching assay. Results are expressed as means ± standard deviation.

Cultivar	Portion	Inhibition Percentage (%)		µg Trolox Equivalents (TE)/g FW		Antioxidant Activity Coefficient (AAC)	
		2021	2022	2021	2022	2021	2022
	Peel	9.22 ^a^ ± 0.15	7.69 ^i^ ± 0.87	148 ^a^ ± 1.81	130 ^i^ ± 10.3	124 ^A^ ± 10.9	134 ^AG^ ± 2.21
*Pardo Lindo*	Seed	5.10 ^b^ ± 0.46	4.15 ^g^ ± 0.22	98.8 ^b^ ± 5.44	87.7 ^g^ ± 2.57	62.7 ^B^ ± 10.9	88.9 ^C^ ± 4.41
	Pulp	0.06 ^c^ ± 0.55	0.62 ^c^ ± 0.00	39.3 ^c^ ± 6.47	45.8 ^c^ ± 0.00	76.8 ^BC^ ± 9.05	64.0 ^B^ ± 4.41
	Peel	14.5 ^d^ ± 0.00	12.5 ^g^ ± 0.65	210 ^d^ ± 0.00	186 ^g^ ± 7.72	89.6 ^C^ ± 12.7	88.9 ^C^ ± 4.41
*Repinau*	Seed	5.42 ^b^ ± 0.31	6.46 ^b^ ± 0.44	103 ^b^ ± 3.63	115 ^b^ ± 5.15	42.3 ^D^ ± 10.9	37.4 ^D^ ± 2.21
	Pulp	0.13 ^c^ ± 0.46	1.85 ^c^ ± 0.00	40.1 ^c^ ± 5.39	60.4 ^c^ ± 0.00	82.0 ^CE^ ± 9.05	51.5 ^BD^ ± 4.41
	Peel	19.2 ^e^ ± 0.15	24.7 ^d^ ± 0.39	266 ^e^ ± 1.82	331 ^d^ ± 4.65	127 ^AF^ ± 10.9	142 ^FG^ ± 4.41
*Pêro Coimbra*	Seed	5.21 ^b^ ± 0.00	6.67 ^bg^ ± 0.79	100 ^b^ ± 0.00	117 ^g^ ± 9.29	147 ^G^ ± 10.9	150 ^G^ ± 2.21
	Pulp	0.00 ^c^ ± 0.00	1.39 ^c^ ± 0.39	33.4 ^c^ ± 7.25	55.0 ^c^ ± 4.65	85.8 ^C^ ± 14.5	70.2 ^BE^ ± 0.00
	Peel	27.8 ^f^ ± 0.61	39.7 ^j^ ± 0.87	367 ^f^ ± 7.25	508 ^j^ ± 10.3	143 ^FG^ ± 9.05	245 ^J^ ± 4.41
*Pêro de Borbela*	Seed	6.72 ^g^ ± 0.92	14.6 ^d^ ± 1.09	118 ^g^ ± 10.9	211 ^d^ ± 12.9	109 ^A^ ± 7.24	130 ^AG^ ± 4.41
	Pulp	0.98 ^c^ ± 0.46	1.38 ^c^ ± 0.22	50.1 ^c^ ± 5.44	54.9 ^c^ ± 2.57	71.7 ^BE^ ± 5.43	87.4 ^C^ ± 6.62
	Peel	53.4 ^h^ ± 1.00	51.5 ^k^ ± 1.96	670 ^h^ ± 11.9	648 ^k^ ± 23.2	283 ^H^ ± 3.62	270 ^H^ ± 8.83
*Noiva*	Seed	13.3 ^d^ ± 0.18	11.5 ^d^ ± 0.65	196 ^d^ ± 2.16	175 ^d^ ± 7.72	196 ^I^ ± 7.24	122 ^A^ ± 6.62
	Pulp	3.93 ^b^ ± 0.36	6.15 ^g^ ± 0.44	85.1 ^b^ ± 4.31	111 ^g^ ± 5.15	76.8 ^BC^ ± 9.05	65.5 ^BE^ ± 6.62

Different letters (a–k) indicate statistically significant differences among IP of 2021 and 2022 or among µg Trolox Equivalents values of 2021 and 2022 (*p* ≤ 0.05). Different letters (A–J) indicate statistically significant differences among AAC of 2021 and 2022 (*p* ≤ 0.05).

**Table 3:** Antioxidant capacity of different portions of pear cultivars using two methods: DPPH radical scavenging assay and the β-carotene bleaching assay. Results are expressed in means ± standard deviation.

Cultivar	Portion	Inhibition Percentage (%)		µg Trolox Equivalent (TE)/g FW		Antioxidant Activity Coefficient (AAC)	
		2021	2022	2021	2022	2021	2022
*Bela-Feia*	Peel	13.2 ^a^ ± 0.31	15.8 ^h^ ± 0.89	195 ^a^ ± 3.63	225 ^h^ ± 10.5	161 ^A^ ± 9.05	178 ^A^ ± 11.0
	Seed	11.9 ^a^ ± 0.31	12.8 ^a^ ± 0.30	180 ^a^ ± 3.63	190 ^a^ ± 3.51	122 ^B^ ± 7.24	90.5 ^C^ ± 6.62
	Pulp	1.63 ^b^ ± 0.15	3.15 ^g^ ± 0.30	57.8 ^b^ ± 1.81	75.8 ^g^ ± 3.51	83.2 ^C^ ± 3.62	68.6 ^CD^ ± 6.62
*Torres Novas*	Peel	7.37 ^c^ ± 0.00	8.19 ^c^ ± 0.30	126 ^c^ ± 0.00	135 ^c^ ± 3.51	88.4 ^C^ ± 3.62	112 ^B^ ± 11.0
	Seed	8.03 ^c^ ± 0.31	7.98 ^c^ ± 0.59	133 ^c^ ± 3.63	133 ^c^ ± 7.03	85.8 ^C^ ± 10.9	76.4 ^CE^ ± 8.83
	Pulp	0.00 ^d^ ± 0.00	0.00 ^d^ ± 0.00	24.5 ^d^ ± 5.44	46.0 ^b^ ± 3.51	64.0 ^D^ ± 9.05	57.7 ^D^ ± 4.41
*Carapinheira Roxa*	Peel	12.6 ^a^ ± 0.00	15.8 ^h^ ± 1.49	187 ^a^ ± 0.00	225 ^h^ ± 17.6	96.0 ^CE^ ± 7.24	109 ^BE^ ± 11.0
	Seed	7.48 ^c^ ± 0.15	7.77 ^c^ ± 0.30	127 ^c^ ± 1.81	130 ^c^ ± 3.51	60.2 ^D^ ± 10.9	49.9 ^D^ ± 2.20
	Pulp	0.11 ^e^ ± 0.46	3.15 ^g^ ± 0.30	39.9 ^e^ ± 5.44	75.8 ^g^ ± 3.51	52.5 ^DF^ ± 10.9	35.9 ^F^ ± 4.41
*Lambe-os-Dedos*	Peel	9.98 ^f^ ± 0.61	12.2 ^a^ ± 0.59	157 ^f^ ± 7.25	183 ^a^ ± 7.03	129 ^B^ ± 7.24	139 ^B^ ± 0.00
	Seed	3.47 ^g^ ± 0.31	9.03 ^cf^ ± 0.89	79.6 ^g^ ± 3.63	145 ^cf^ ± 10.54	39.7 ^F^ ± 10.9	67.1 ^CD^ ± 8.83
	Pulp	0.22 ^e^ ± 0.61	3.57 ^g^ ± 0.89	41.1 ^e^ ± 7.25	80.8 ^g^ ± 10.54	60.2 ^D^ ± 7.24	62.4 ^D^ ± 3.54
*Amorim*	Peel	15.4 ^h^ ± 1.31	13.7 ^ah^ ± 1.89	221 ^h^ ± 3.63	200 ^ah^ ± 10.54	216 ^G^ ± 7.24	228 ^G^ ± 2.21
	Seed	12.9 ^a^ ± 0.15	14.5 ^ah^ ± 0.89	191 ^a^ ± 1.81	210 ^ah^ ± 10.54	152 ^A^ ± 10.9	175 ^A^ ± 6.62
	Pulp	8.57 ^c^ ± 0.46	8.19 ^c^ ± 0.89	140 ^c^ ± 5.44	135 ^c^ ± 10.54	76.8 ^CD^ ± 9.05	98.3 ^C^ ± 4.41
*Carapinheira*	Peel	14.8 ^h^ ± 0.00	- *	213 ^h^ ± 0.00	- *	163 ^A^ ± 7.24	- *
	Seed	21.4 ^i^ ± 0.15	- *	291 ^i^ ± 1.81	- *	165 ^A^ ± 7.24	- *
	Pulp	0.65 ^b^ ± 0.31	- *	46.3 ^b^ ± 3.63	- *	41.0 ^F^ ± 9.05	- *

* The authors did not had access to the 2022 harvest of the *Carapinheira* pear cultivar. Different letters (a–k) indicate statistically significant differences among IP of 2021 and 2022 or among µg Trolox Equivalents values of 2021 and 2022 harvest years (*p* ≤ 0.05). Different letters (A–J) indicate statistically significant differences among AAC of 2021 and 2022 harvest years (*p* ≤ 0.05).

**Table 4:** Total Phenolics Content (TPC) and Total Flavonoids Content (TFC) of different portions of apple cultivars. Results are expressed in means ± standard deviation.

Cultivar	Portion	Total Phenolics Content (µg GAE/g FW)		Total Flavonoids Content (µg EE/g FW)	
		2021	2022	2021	2022
	Peel	846 ^a^ ± 13.1	748 ^j^ ± 2.19	295 ^A^ ± 0.00	427 ^I^ ± 12.4
*Pardo Lindo*	Seed	587 ^b^ ± 12.0	298 ^e^ ± 1.09	211 ^B^ ± 4.14	246 ^AB^ ± 12.4
	Pulp	366 ^c^ ± 1.09	149 ^k^ ± 2.19	43.7 ^C^ ± 0.00	35.0 ^C^ ± 4.14
	Peel	962 ^d^ ± 21.9	744 ^j^ ± 2.19	562 ^D^ ± 12.4	579 ^D^ ± 20.7
*Repinau*	Seed	598 ^b^ ± 19.7	276 ^e^ ± 2.19	262 ^A^ ± 8.28	225 ^B^ ± 16.6
	Pulp	326 ^ce^ ± 3.28	127 ^k^ ± 2.19	37.9 ^C^ ± 0.00	37.9 ^C^ ± 0.00
	Peel	1207 ^f^ ± 27.3	1146 ^l^ ± 49.2	749 ^E^ ± 12.4	667 ^J^ ± 29.0
*Pêro Coimbra*	Seed	623 ^b^ ± 13.1	875 ^a^ ± 8.74	360 ^F^ ± 0.00	237 ^B^ ± 24.8
	Pulp	291 ^e^ ± 0.00	276 ^e^ ± 26.2	70.1 ^C^ ± 4.14	17.4 ^C^ ± 12.4
	Peel	1424 ^g^ ± 43.7	1651 ^m^ ± 43.7	778 ^G^ ± 12.4	1205 ^K^ ± 20.7
*Pêro de Borbela*	Seed	627 ^b^ ± 5.46	700 ^i^ ± 2.19	228 ^B^ ± 4.14	497 ^L^ ± 4.14
	Pulp	294 ^e^ ± 0.00	310 ^e^ ± 12.0	37.9 ^C^ ± 0.00	64.2 ^C^ ± 4.14
	Peel	1964 ^h^ ± 0.00	1801 ^n^ ± 38.2	1284 ^H^ ± 0.00	1389 ^M^ ± 24.8
*Noiva*	Seed	690 ^i^ ± 2.19	627 ^b^ ± 3.28	357 ^F^ ± 4.14	313 ^A^ ± 16.6
	Pulp	261 ^e^ ± 1.09	275 ^e^ ± 1.09	64.2 ^C^ ± 4.14	84.7 ^C^ ± 8.28

Different letters (a–n) indicate statistically significant differences among Total Phenolics of 2021 and 2022 harvest years (*p* ≤ 0.05). Different letters (A–M) indicate statistically significant differences among Total Flavonoids of 2021 and 2022 harvest years (*p* ≤ 0.05).

**Table 5:** TPC and TFC of different portions of pear cultivars. Results are expressed in means ± standard deviation.

Cultivar	Portion	Total Phenolics Content (µg GAE/g)		Total Flavonoids Content (µg EE/g)	
		2021	2022	2021	2022
	Peel	948 ^a^ ± 25.1	1027 ^a^ ± 74.3	389 ^A^ ± 8.28	418 ^A^ ± 24.8
*Bela-Feia*	Seed	778 ^b^ ± 24.0	1399 ^e^ ± 238	155 ^B^ ± 8.28	196 ^B^ ± 41.4
	Pulp	339 ^c^ ± 2.19	199 ^g^ ± 73.2	11.6 ^C^ ± 20.7	5.70 ^C^ ± 12.4
	Peel	862 ^a^ ± 12.0	783 ^b^ ± 18.6	339 ^D^ ± 12.4	430 ^A^ ± 57.9
*Torres Novas*	Seed	632 ^d^ ± 7.65	474 ^d^ ± 9.83	216 ^E^ ± 4.14	208 ^E^ ± 24.8
	Pulp	280 ^c^ ± 6.56	187 ^g^ ± 39.3	0.00 ^C^ ± 0.00	0.00 ^C^ ± 0.00
	Peel	991 ^a^ ± 32.8	1139 ^f^ ± 44.8	257 ^E^ ± 12.4	319 ^DF^ ± 8.28
*Carapinheira Roxa*	Seed	484 ^d^ ± 13.1	500 ^d^ ± 50.3	111 ^B^ ± 12.4	114 ^B^ ± 16.6
	Pulp	306 ^c^ ± 1.09	237 ^c^ ± 21.9	0.00 ^C^ ± 0.00	0.00 ^C^ ± 0.00
	Peel	731 ^bd^ ± 19.7	1064 ^af^ ± 44.8	292 ^F^ ± 12.4	354 ^AD^ ± 24.8
*Lambe-os-Dedos*	Seed	466 ^c^ ± 17.5	511 ^d^ ± 108	126 ^B^ ± 0.00	137 ^B^ ± 8.28
	Pulp	299 ^c^ ± 7.65	170 ^g^ ± 30.6	0.00 ^C^ ± 0.00	0.00 ^C^ ± 0.00
	Peel	1409 ^e^ ± 40.4	1095 ^f^ ± 43.7	547 ^G^ ± 33.1	541 ^G^ ± 41.4
*Amorim*	Seed	865 ^ab^ ± 25.1	500 ^d^ ± 80.9	237 ^E^ ± 16.6	158 ^B^ ± 12.4
	Pulp	367 ^c^ ± 4.37	197 ^g^ ± 29.5	23.3 ^C^ ± 4.14	26.2 ^C^ ± 8.28
	Peel	1102 ^f^ ± 35.0	- *	371 ^A^ ± 24.8	- *
*Carapinheira*	Seed	1164 ^f^ ± 39.3	- *	448 ^D^ ± 8.28	- *
	Pulp	376 ^c^ ± 4.37	- *	61.3 ^H^ ± 0.00	- *

* The authors did not had access to the 2022 harvest of the *Carapinheira* pear cultivar. Different letters (a–g) indicate statistically significant differences among Total Phenolics of 2021 and 2022 harvest years (*p* ≤ 0.05). Different letters (A–G) indicate statistically significant differences among Total Flavonoids of 2021 and 2022 harvest years (*p* ≤ 0.05).

**Table 6:** Total fructose content of different portions of apple and pear cultivars. Results are expressed in means ± standard deviation.

Apple Cultivar	Portion	Total Fructose Content (mg Fructose/g FW)		Pear Cultivar	Portion	Total Fructose Content (mg Fructose/g FW)	
		2021	2022			2021	2022
	Peel	95.0 ^a^ ± 4.61	96.0 ^a^ ± 4.17		Peel	48.3 ^A^ ± 3.95	47.7 ^A^ ± 2.20
*Pardo Lindo*	Seed	84.6 ^b^ ± 5.71	82.8 ^b^ ± 3.95	*Bela-Feia*	Seed	57.6 ^AB^ ± 4.83	62.4 ^BC^ ± 5.05
	Pulp	107 ^a^ ± 5.05	110 ^a^ ± 2.85		Pulp	56.5 ^AB^ ± 5.93	52.4 ^A^ ± 4.39
	Peel	89.6 ^a^ ± 4.83	87.4 ^c^ ± 3.51		Peel	57.3 ^AB^ ± 4.39	60.0 ^BC^ ± 3.73
*Repinau*	Seed	71.8 ^a^ ± 5.05	74.6 ^a^ ± 3.73	*Torres Novas*	Seed	68.8 ^C^ ± 4.83	66.9 ^BC^ ± 3.95
	Pulp	88.5 ^a^ ± 4.61	89.0 ^ac^ ± 5.27		Pulp	44.3 ^A^ ± 3.07	39.2 ^A^ ± 4.61
	Peel	97.7 ^a^ ± 3.95	101 ^a^ ± 4.61		Peel	60.4 ^BC^ ± 4.39	62.6 ^BC^ ± 4.39
*Pêro Coimbra*	Seed	94.0 ^a^ ± 6.15	92.4 ^a^ ± 2.20	*Carapinheira Roxa*	Seed	66.0 ^BC^ ± 3.51	66.0 ^BC^ ± 5.71
	Pulp	95.8 ^c^ ± 5.71	97.5 ^c^ ± 2.41		Pulp	63.7 ^BC^ ± 5.05	57.6 ^AB^ ± 3.95
	Peel	89.6 ^a^ ± 3.95	84.2 ^a^ ± 4.75		Peel	48.9 ^A^ ± 4.39	51.9 ^A^ ± 5.05
*Pêro de Borbela*	Seed	75.2 ^b^ ± 5.49	82.8 ^a^ ± 6.19	*Lambe-os-Dedos*	Seed	49.3 ^A^ ± 4.39	51.4 ^A^ ± 6.15
	Pulp	107 ^c^ ± 4.17	102 ^c^ ± 2.55		Pulp	57.9 ^A^ ± 5.27	56.2 ^A^ ± 5.49
	Peel	73.6 ^b^ ± 5.05	79.0 ^b^ ± 3.75		Peel	69.9 ^C^ ± 3.73	75.2 ^D^ ± 2.85
*Noiva*	Seed	71.3 ^b^ ± 5.71	82.5 ^a^ ± 3.99	*Amorim*	Seed	87.6 ^D^ ± 2.41	88.4 ^D^ ± 3.51
	Pulp	85.6 ^a^ ± 6.59	94.9 ^a^ ± 4.92		Pulp	51.1 ^A^ ± 2.20	61.4 ^BC^ ± 2.63
					Peel	46.8 ^A^ ± 3.95	- *
				*Carapinheira*	Seed	51.3 ^A^ ± 4.61	- *
					Pulp	68.8 ^C^ ± 4.39	- *

* The authors did not had access to the 2022 harvest of the *Carapinheira* pear cultivar. Different letters (a–c) indicate statistically significant differences among Total Fructose content of apple cultivars of 2021 and 2022 harvest years (*p* ≤ 0.05). Different letters (A–D) indicate statistically significant differences among Total Fructose content of pear cultivars of 2021 and 2022 harvest years (*p* ≤ 0.05).

**Table 7:** Linearity, sensitivity and recovery percentages for the UHPLC-ToF-MS method. (n.d. = not determined).

Standard	rt (min)	Equation	r^2^	Recovery Percentage		Linear Range (µg/mL)	LOQ (µg/g)	LOD (µg/g)
				Spiking Level 0.1 (mg/100 g)	Spiking Level 1.0 (mg/100 g)			
4-Hydroxybenzoic Acid	3.34	y = 329,563x + 21,605	0.9805	92.0	97.3	0.25–10	2.5	1.0
Apigenin	6.21	y = 4 × 10^7^+ 89,331	0.9993	85.1	77.1	0.0025–5	0.025	0.01
Caffeic Acid	3.52	y = 385,020x + 7627	0.9960	95.8	87.9	0.5–10	5.0	2.5
Catechin	3.34	y = 3 × 10^6^ + 105,538	0.9933	75.2	76.9	0.25–10	2.5	1.0
Chlorogenic Acid	3.23	y = 393,929x + 7739.1	0.9929	n.d.	78.3	0.25–10	2.5	1.0
Epicatechin	3.59	y = 3 × 10^6^ + 53,261	0.9935	72.4	83.3	0.1–10	1.0	0.5
Eriodyctiol	5.53	y = 8 × 10^6^ + 27,573	0.9986	84.3	87.8	0.005–10	0.05	0.025
Gallic Acid	1.18	y = 90,742x + 6145.5	0.9814	82.5	87.7	0.01–10	0.1	0.05
Luteolin	5.71	y = 2 × 10^7^ + 288,365	0.9937	87.0	75.2	0.025–5	0.25	0.1
Naringenin	6.06	y = 5 × 10^6^ + 5148.3	0.9994	80.6	87.0	0.25–10	2.5	1.0
*o*-Coumaric Acid	4.86	y = 246,146x + 1871.2	0.9905	85.6	88.2	0.5–10	5.0	2.5
*p*-Coumaric Acid	4.11	y = 261,740x + 10,462	0.9923	81.5	87.5	0.5–10	5.0	2.5
Phlorizin	4.88	y = 49,825x + 1778.4	0.9915	75.4	85.8	0.025–5	0.25	0.1
Quercetin	5.79	y = 4 × 10^6^ + 5067.2	0.9983	84.4	86.7	0.025–10	0.25	0.1
Quercetin-3-B-D-Glucoside	4.32	y = 3 × 10^6^ + 4105.4	0.9969	94.0	88.1	0.025–10	0.25	0.1
Quercitrin	4.62	y = 1 × 10^6^ + 1287.9	0.9992	77.5	85.4	0.025–10	0.25	0.1
Rutin	4.13	y = 1 × 10^6^ + 3288.2	0.9946	80.5	87.1	0.25–10	2.5	1.0
Sinapic Acid	4.14	y = 415,150x + 11,493	0.9960	82.2	86.5	0.25–5	2.5	1.0
Syringic Acid	3.48	y = 492,820x + 5813.3	0.9957	70.2	88.5	1.0–10	10.0	5.0
*trans*-Ferulic Acid	4.24	y = 418,556x + 1616.9	0.9953	n.d.	82.2	1.0–10	10.0	5.0
Vanillic Acid	3.49	y = 287,981x + 31,116	0.9853	76.0	88.9	1.0–10	10.0	5.0

**Table 8:** Results of the relative standard deviation repeatability (RSD_r_) and relative standard deviation of inter-day precision (RSD_R_) at different spiking levels.

Standard	Spiking Level	RSD_r_ (%)	RSD_R_ (%)	Standard	Spiking Level	RSD_r_ (%)	RSD_R_ (%)
4-Hydroxybenzoic Acid	0.1 mg/100 g	2.50 1.63 1.53	0.78	*p*-Coumaric Acid	0.1 mg/100 g	10.1 4.37 6.08	10.2
	1.0 mg/100 g	11.0 4.97 2.65	3.03		1.0 mg/100 g	3.33 2.74 2.88	4.39
Apigenin	0.1 mg/100 g	2.18 1.55 1.38	7.35	Phlorizin	0.1 mg/100 g	4.34 2.23 5.89	5.93
	1.0 mg/100 g	2.62 1.32 0.74	4.10		1.0 mg/100 g	4.72 1.69 1.12	6.34
Caffeic Acid	0.1 mg/100 g	12.2 7.87 8.88	23.8	Quercetin	0.1 mg/100 g	5.71 3.74 4.08	3.73
	1.0 mg/100 g	3.60 3.03 3.53	4.89		1.0 mg/100 g	2.96 2.84 1.47	4.61
Catechin	0.1 mg/100 g	4.01 1.56 1.99	3.82	Quercetin-3-B-D-Glucoside	0.1 mg/100 g	5.52 2.84 3.78	4.38
	1.0 mg/100 g	6.39 1.35 2.61	5.37		1.0 mg/100 g	3.40 2.11 1.16	4.79
Chlorogenic Acid	0.1 mg/100 g	6.33 1.27 2.40	4.52	Quercitrin	0.1 mg/100 g	8.51 1.94 3.97	13.3
	1.0 mg/100 g	3.97 1.73 1.51	6.56		1.0 mg/100 g	1.83 1.49 1.05	5.37
Epicatechin	0.1 mg/100 g	2.78 2.04 1.74	5.39	Rutin	0.1 mg/100 g	7.65 4.60 4.73	5.88
	1.0 mg/100 g	5.48 6.60 1.70	3.49		1.0 mg/100 g	4.52 2.88 2.35	4.35
Eriodyctiol	0.1 mg/100 g	6.38 2.43 2.21	5.17	Sinapic Acid	0.1 mg/100 g	6.97 5.07 2.98	4.42
	1.0 mg/100 g	4.30 2.40 1.62	5.50		1.0 mg/100 g	3.56 5.47 2.21	6.91
Gallic Acid	0.1 mg/100 g	2.10 7.18 6.25	14.2	Syringic Acid	0.1 mg/100 g	5.90 2.50 2.25	8.78
	1.0 mg/100 g	10.8 16.6 6.48	10.2		1.0 mg/100 g	3.40 2.00 1.69	5.62
Luteolin	0.1 mg/100 g	3.51 1.49 1.90	7.54	*trans*-Ferulic Acid	0.1 mg/100 g	11.4 6.42 5.67	8.93
	1.0 mg/100 g	3.91 1.35 1.77	5.99		1.0 mg/100 g	4.67 1.33 3.15	4.20
Naringenin	0.1 mg/100 g	22.5 14.9 12.9	20.1	Vanillic Acid	0.1 mg/100 g	12.3 7.28 10.6	9.26
	1.0 mg/100 g	9.74 3.08 1.27	7.61		1.0 mg/100 g	3.37 3.37 1.69	5.51
*o*-Coumaric Acid	0.1 mg/100 g	5.93 6.69 5.93	6.81				
	1.0 mg/100 g	2.32 2.05 2.27	6.07				

**Table 9:** Phenolic compounds (µg/g FW) in different parts of apple regional cultivars (P: Peel, S: Seed, M: Mesocarp (pulp)).

Phenolic Compound	*Pardo Lindo*			*Repinau*			*Pêro Coimbra*			*Pêro de Borbela*			*Noiva*		
	P	S	M	P	S	M	P	S	M	P	S	M	P	S	M
4-Hydroxybenzoic Acid	2.226	-	-	2.112	<LOQ	-	12.37	1.025	-	40.98	27.10	1.119	36.35	8.274	1.688
Apigenin	<LOQ	<LOQ	-	-	<LOQ	-	-	<LOQ	-	-	-	-	-	<LOQ	-
Caffeic Acid	-	-	-	-	-	-	-	-	-	-	-	-	-	-	-
Catechin	-	-	-	3.536	-	-	-	-	-	4.508	5.389	-	5.986	0.612	-
Chlorogenic Acid	34.92	9.955	3.189	1.062	6.155	0.610	65.84	-	9.003	43.07	293.5	10.42	123.6	183.9	55.21
Epicatechin	<LOQ	4.590	<LOQ	4.495	0.824	<LOQ	25.84	-	<LOQ	96.11	59.31	3.095	80.70	16.37	3.745
Eriodyctiol	0.010	0.014	-	-	0.050	-	0.010	<LOQ	<LOQ	-	-	-	-	-	-
Gallic Acid	5.445	4.126	3.781	4.785	5.535	4.079	2.756	1.867	2.320	4.530	5.608	4.539	6.876	6.715	7.857
Luteolin	<LOQ	<LOQ	-	-	-	-	<LOQ	-	<LOQ	-	-	-	-	-	-
Naringenin	0.078	0.052	-	0.074	0.078	-	0.346	0.158	0.033	-	-	-	-	-	-
o-Coumaric Acid	6.099	5.904	-	-	12.61	-	2.504	-	-	16.16	23.67	0.634	7.350	6.440	-
*p*-Coumaric Acid	<LOQ	<LOQ	-	-	3.394	-	2.368	-	-	15.46	10.46	-	6.158	1.908	-
Phlorizin	11.36	33.36	-	2.704	58.82	<LOQ	23.11	13.37	1.364	137.8	318.7	9.778	67.26	86.40	4.165
Quercetin	2.479	-	-	-	0.096	-	0.514	-	-	2.295	0.254	-	0.180	<LOQ	-
Quercetin-3-B-D-Glucoside	4.647	0.991	-	23.96	1.741	-	49.69	1.708	0.117	154.4	20.27	0.293	9.352	0.923	0.064
Quercitrin	16.08	2.323	0.476	20.27	2.702	-	59.52	9.973	1.251	-	8.793	2.914	1.540	0.859	0.406
Rutin	0.595	0.093	-	2.154	0.099	-	4.122	-	-	87.24	4.053	-	3.707	0.376	-
Sinapic Acid	-	-	-	-	-	-	-	-	-	-	-	-	-	-	-
Syringic Acid	-	-	-	-	-	-	-	-	-	-	-	-	-	-	-
trans-Ferulic Acid	1.090	<LOQ	-	<LOQ	1.516	-	1.108	3.829	<LOQ	<LOQ	-	-	-	-	-
Vanillic Acid	8.997	4.092	<LOQ	6.863	4.989	1.427	9.957	5.739	2.357	9.181	5.495	<LOQ	6.153	5.587	2.546
**SUM**	**94.02**	**65.50**	**7.447**	**72.01**	**98.61**	**6.116**	**260.1**	**37.67**	**16.45**	**611.8**	**782.6**	**32.79**	**355.2**	**318.4**	**75.68**

**Table 10:** Phenolic compounds (µg/g FW) in different parts of pear regional cultivars (P: Peel, S: Seed, M: Mesocarp (pulp)).

Phenolic Compound	*Bela Feia*			*Torres Novas*			*Carapinheira Roxa*			*Lambe-os-Dedos*			*Amorim*			*Carapinheira*		
	P	S	M	P	S	M	P	S	M	P	S	M	P	S	M	P	S	M
4-Hydroxybenzoic Acid	4.679	4.151	-	2.193	-	-	16.59	1.894	-	17.05	8.045	-	13.65	4.042	-	16.10	13.57	-
Apigenin	-	-	-	-	-	-	-	-	-	-	-	-	-	-	-	-	-	-
Caffeic Acid	14.53	-	-	23.84	9.508	3.994	17.27	3.835	-	22.80	-	-	27.09	8.777	-	15.80	5.894	2.574
Catechin	0.620	1.224	-	-	2.530	-	4.263	1.992	-	2.781	2.173	-	2.358	0.263	-	1.782	2.579	<LOQ
Chlorogenic Acid	46.36	21.40	-	103.0	123.1	33.29	277.5	32.83	45.17	118.0	118.3	65.12	63.23	66.87	75.13	340.2	156.7	21.95
Epicatechin	8.953	8.782	-	0.190	5.100	-	36.85	4.567	0.683	37.30	14.90	0.192	29.61	9.053	0.312	30.87	28.21	0.760
Eriodyctiol	0.095	-	-	0.036	0.059	-	-	-	-	<LOQ	-	-	<LOQ	-	-	<LOQ	-	-
Gallic Acid	-	1.708	-	-	-	-	2.836	-	3.037	2.131	-	5.802	-	-	-	2.803	4.454	3.408
Luteolin	-	-	-	-	<LOQ	-	-	-	-	<LOQ	-	-	-	-	-	-	-	-
Naringenin	0.329	0.028	-	0.192	0.112	-	0.110	<LOQ	<LOQ	0.122	<LOQ	-	0.130	<LOQ	-	0.062	<LOQ	-
o-Coumaric Acid	-	-	-	1.015	0.748	-	-	-	-	-	-	-	-	-	-	-	-	-
*p*-Coumaric Acid	2.469	-	-	-	4.067	-	-	-	-	0.971	-	-	-	-	-	-	-	-
Phlorizin	-	-	2.605	-	-	-	2.139	-	-	-	-	-	-	-	0.792	4.445	5.753	4.904
Quercetin	0.861	0.109	-	-	0.978	-	0.826	0.191	-	0.350	<LOQ	-	0.530	-	-	0.508	0.044	-
Quercetin-3-B-D-Glucoside	14.68	1.085	<LOQ	22.27	1.406	0.028	5.580	0.531	0.066	4.751	0.770	0.052	7.868	0.290	<LOQ	19.76	1.296	<LOQ
Quercitrin	1.432	0.176	-	5.333	0.403	-	0.915	0.179	-	1.264	0.330	-	3.100	0.152	-	4.801	0.488	0.212
Rutin	0.307	-	-	32.60	0.762	-	1.611	0.060	-	-	0.163	-	0.453	0.124	-	59.61	4.979	-
Sinapic Acid	-	-	-	-	-	-	-	-	-	-	-	-	1.078	0.612	-	-	-	-
Syringic Acid	2.198	1.438	1.309	-	1.322	0.519	7.897	3.917	2.080	5.167	6.036	3.348	4.297	5.761	3.472	1.805	1.307	-
trans-Ferulic Acid	1.351	1.533	<LOQ	-	1.680	-	1.936	<LOQ	-	1.659	2.932	1.669	1.575	3.921	1.092	-	<LOQ	-
Vanillic Acid	4.486	-	3.482	13.37	-	5.012	25.69	7.677	9.662	15.39	29.49	22.67	11.65	69.36	24.85	18.88	13.47	5.571
**SUM**	**103.3**	**41.64**	**7.397**	**204.0**	**151.7**	**42.84**	**402.0**	**57.67**	**60.69**	**229.7**	**183.1**	**98.85**	**166.6**	**169.2**	**105.7**	**517.4**	**238.8**	**39.37**

## Data Availability

Data sharing is not applicable to this article.

## Supplementary Materials

The following supporting information can be downloaded at: https://www.mdpi.com/article/10.3390/foods12071537/s1, Figure S1: Photos of the traditional cultivars of apples and pears selected for the study.
