# Supplementary material for: Antioxidant Capacity and Phenolics Profile of Portuguese Traditional Cultivars of Apples and Pears and Their By-Products: On the Way to Newer Applications
Source: Foods. 2023 Apr 5;12(7):1537. doi: 10.3390/foods12071537 (PMC10094612; doi:10.3390/foods12071537)
Supplement: Supplementary file 1 [file foods-12-01537-s001.zip › foods-2306258-supplementary.pdf]

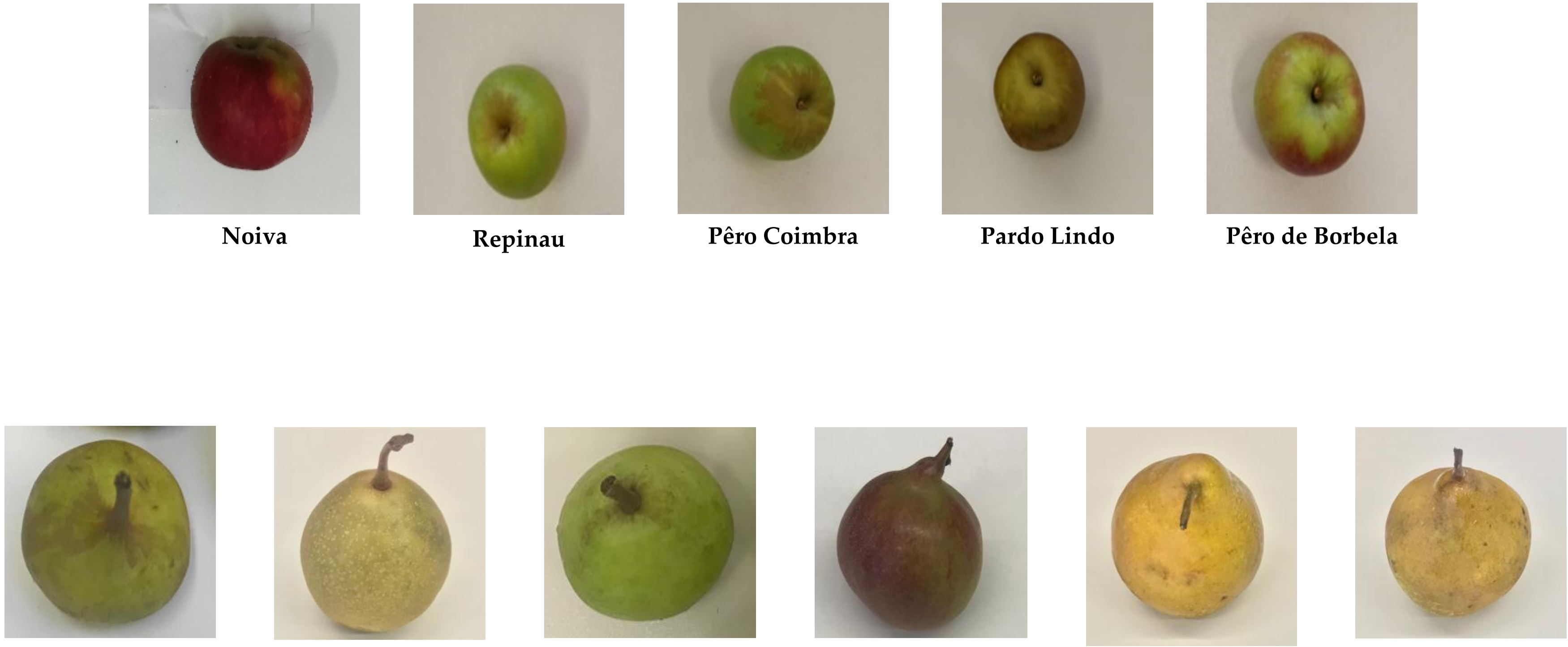

Figure S1: Photos of the traditional cultivars of apples and pears selected for the study.
